# Effect of feed additive “Ceobalyk” on the biological and microbiological parameters of African sharptooth catfish (*Clarias gariepinus*)

**DOI:** 10.14202/vetworld.2021.669-677

**Published:** 2021-03-19

**Authors:** Nurzhan Biltebaevna Sarsembayeva, Ardak Sabyrzhanovna Akkozova, Tolkyn Bakytovna Abdigaliyeva, Aida Bolatbekovna Abzhalieva, Aray Berkimbekovna Aidarbekova

**Affiliations:** 1 Kazakh National Agrarian University, 8 Abay Avenue, Almaty, 050010, Republic of Kazakhstan; 2 Almaty Technological University, 100 Tole Bi Street, Almaty, 050012, Republic of Kazakhstan

**Keywords:** African sharptooth catfish, *Escherichia coli*, feed additives, microbiological parameters, quality, total viable count

## Abstract

**Aim:**

This study aimed to analyze biological and microbiological parameters of *Clarias gariepinus* bred at Chundzha natural hot spring in the Almaty region of Kazakhstan, a new feed additive, namely, the prebiotic “Ceobalyk.”

**Materials and Methods:**

Two groups (50 heads each) of fish of the same age were established and kept in specialized rectangular pools of AsylTasEngineering LLP. The experiment lasted 61 days. We used the feed additive “Ceobalyk”, developed based on natural minerals. Feed additive was added to the feed of the experimental group of fish in an amount of 10% per 1 kg of the main diet. Laboratory studies were conducted at the laboratories of the Kazakhstan-Japan Innovation Center. The quality of the fish was studied in accordance with the standards of the Republic of Kazakhstan and interstate standards.

**Results:**

In biological and microbiological studies of all samples of African sharptooth catfish, which received a new type of feed additive “Ceobalyk”, the pH values of fish meat in the experimental groups were normal and varied in the range from 6.5 to 6.7. When setting up the reaction with copper sulfate, the reaction was positive. During bacterioscopy, there was a noticeable decrease in the number of microbes (2-3 bacteria less) in comparison with the control. An increase in the indices of the absolute body length and body width was observed by the end of the study. The body length in the experimental group was significantly greater (by 2.12% on average) than that in the control group. In the experimental variant, the average weight of fish was 21.8% higher. As a result of organoleptic studies, it was revealed that the musculature of the fish of the experimental groups was dense and elastic; when pressed on the skin with a finger, a fossa did not remain; the smell was specific, fresh. When tested by cooking, the broth was transparent and aromatic.

**Conclusion:**

This feed additive “Ceobalyk” does not cause deviations in the physiological status of fish and can be used as part of the main diet.

## Introduction

Kazakhstan has a rich fishery water fund and favorable conditions for the intensive development of fish farming. Considering the projected increase in the population and, based on the norm recommended by science (14.6 kg per person), to meet the population’s need for fish and fish products, it is necessary to bring the volume of catch, growing marketable fish, and import of fish to 272.0 thousand tons per year. Currently, one of the main tasks of the agro-industrial complex is to meet the needs of the population in fish products of the required assortment, high quality, and at affordable prices. This is impossible without increasing the productivity of valuable fish species [1-3]. In recent years, the global supply of fish for human consumption has outpaced population growth [4]. Private commercial fish farming currently shows active growth through investment subsidies for research projects in the field of recirculated water supply and cage farms [5,6].

Breeding of clariids is one of the oldest forms of fish farming in South and Southeast Asia, as well as Africa [7]. African sharptooth catfish is significantly superior in taste to Kazakh catfish species. Ichthyologists place sharptooth catfish on a par with sturgeons, salmonid fishes, and eel in terms of the consistency and nutritional value of the meat [8,9]. The tender white meat of the fish contains practically no small bones, which makes it possible to use it in baby food. Due to the optimal combination of proteins, fats, and amino acids (necessary for a healthy diet), Clary catfish meat is classified as a dietary product [8,9]. Sharptooth catfish meat is rich in omega-3 fatty acids, the level of which is even higher than in mackerel and rainbow trout. These acids are among the most valuable components of fish oil: They relieve inflammation during arthritis, ameliorate chronic fatigue syndrome, prevent atherosclerosis and heart diseases, lower blood cholesterol, and promote the development of the brain and nervous system in children [8,9].

Recently, industrial fish breeding in cold climates using recirculating aquaculture systems has become increasingly popular, which allows stable and optimal conditions for high productivity to be achieved. The use of high-quality feed developed based on scientific work in the field of fish farming will allow obtaining and certifying safe organic food from fish [10-12].

Feed for such systems should have high nutritional value and quality, as well as optimal technological properties, namely, high stability in water and firmness of granules during feeding [13,14]. Numerous studies have been performed on the use of zeolites in veterinary practice and animal husbandry. The first of these testing zeolites in fish farming showed the possibility of their successful use as a feed additive [15]. It is anticipated that the use of zeolites and other aluminosilicates of heulandite zeolites in feed for fish can confer significant benefits.

The addition of natural zeolites such as clinoptilolite to compound feed at low doses of about 1-2% has a positive effect on the physiological state of the body of animals and birds [16].

The use of the “Ceobalyk” feed additive in the diet of fish improves nutrient utilization, promotes the intensive growth of young animals, increases the productivity of adult fish, prevents a number of gastrointestinal diseases, and improves the quality of meat and scales [17]. The “Ceobalyk” feed additive is a product of the processing of environmentally friendly natural zeolite hassock from the Chankanai deposit (Kazakhstan) crushed to fragments of 1-4 mm in size. The presence of macro- and micronutrients vital for the organism (iron, zinc, copper, magnesium, calcium, and potassium) makes this product an essential component of feed mixtures since microelements are of fundamental importance for maintaining health, growth, and many biochemical and physiological functions of the body of animals, including fish [18,19]. The “Ceobalyk” feed additive is non-toxic, non-radioactive, non-combustible, and non-explosive [20]. “Ceobalyk” meets the quality requirements specified in Table-1.

**Table-1:** Specifications of the “Ceobalyk” feed additive.

No	Parameter	Specifications and normal values
1.	Appearance	Light pink and pink microgranules
2.	Smell	No smell
3.	Mass fraction of clinoptilolite, not less than (%)	50
4.	Mass fraction of moisture, not more than (%)	10
5.	Grinding coarseness Oversize, mesh aperture 4.0 mm, not more than (%)	5
	Undersize, mesh aperture 1.0 mm, not more than (%)	5
6.	Mass fraction of metal foreign matter, not more than (%)	0.01
7.	Mass fraction of:	
7.1.	Fluorine, not more than (%)	0.15
7.2.	Arsenic, not more than (%)	0.003
7.3.	Lead, not more than (%)	0.002
7.4.	Mercury, not more than (%)	0.0005
7.5.	Cadmium, not more than (%)	0.05

The aim of this study was to determine the value for fish breeding, and biological and microbiological properties of using the feed additive Ceobalyk” in the diet of African sharptooth catfish.

## Materials and Methods

### Ethical approval

The Bioethics Commission of the nonprofit joint stock company Kazakh National Agrarian University approved this study. The experiments and the used fish research methodology were in accordance with the biological safety requirements and ethical principles of animal experimentation stated in the European Convention for the Protection of Vertebrate Animals used for Experimental and Other Scientific Purposes.

### Study period and location

**Figure-1:**
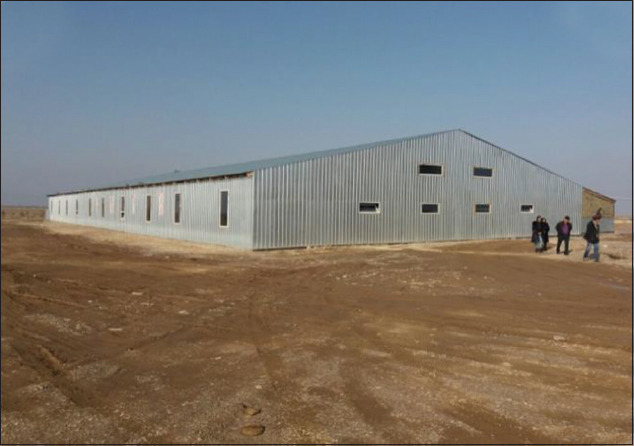
The Tengryfish LLP site.

The experimental studies on fish feeding were carried out from March to May 2019 at the fish farm of the TENGRYFISH LLP, located in Chundzha natural hot spring in the Enbekshikazakh district of the Almaty region, Kazakhstan (Figure-1).

**Figure-2:**
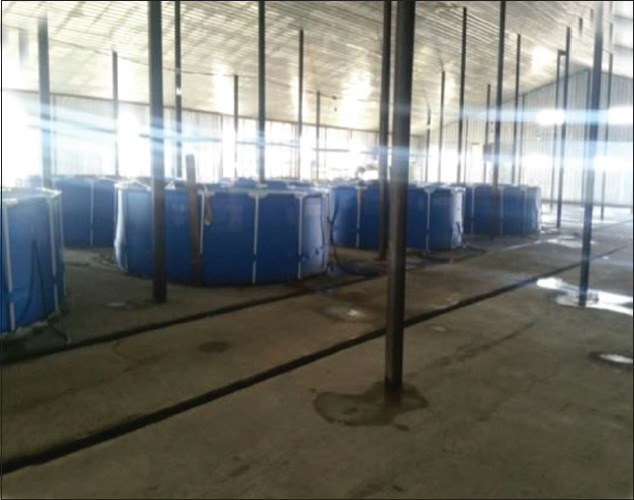
Pools in the production facility.

The fish farm is located 270 km from Almaty, near the village of Chundzha, on a 15 ha plot of private land where there are two deep-flowing artesian geothermal wells. Water with a temperature of +29°C (year-round) is supplied from a depth of 650 m; the total water flow is over 40 L/s (Figure-2). A fish breeding production facility with a total area of 1620 m^2^ was built on the site. A flow-through water supply system with a total pool volume of 750 m3 is used in the facility.

### Study objects

The subjects of this research were commercial specimens of African sharptooth catfish (*Clarias gariepinus*), to the feed of which a feed additive “Ceobalyk” was added. It was developed during the AsylTasEngineering LLP scientific project for the “Production of organic fish food (Tilapia, African sharptooth catfish, etc.) bred using local ecologically clean feed in accordance with international standards.”

Two groups with 50 fish in each were established for the study. The fish were kept in artificial pools with an average water temperature of 23°C. The experiments were carried out in accordance with the research scheme presented in Table-2.

**Table-2:** Experiment scheme.

Group No.	Number of fish	Conditions
African sharptooth catfish		
Control	50	F (100%)
Experimental	50	F (90%) + FA (10%)

F=Feed, FA=Feed additive “Ceobalyk”

The initial weight at seeding was 179±0.9 g in the experimental group and 181±0.3 g in the control group. The duration of the experiment was 61 days. The maintenance conditions and hydrological and temperature water regimes were the same for all experimental and control groups of fish.

###  Composition of the “Ceobalyk” feed additive

A new type of feed additive was used in the study. The use of this additive provides a balanced feed composition, allows efficient feeding of commercial sharptooth catfish weighing from 100 to 500 g, increases the feed’s energy value, and facilitates long-term storage until use. The advantage of the feed additive is the fact that, in addition to the normal ingredients of compound feed for commercial 100-500 g sharptooth catfish (fish meal, tankage, dried blood, soybean meal, fodder yeast, and premix), it also includes fish oil, soybean oil, corn gluten, wheat, preservatives, and antioxidants. The average proportions of components are as follows (wt. %): Fish meal – 22.00; tankage – 12.00; dried blood – 5.00; soybean meal – 13.00; fodder yeast – 14.00; fish oil – 3.00; soybean oil – 3.20; corn gluten – 7.00; wheat – 19.70; premix – 1.00; preservatives – 0.05; and antioxidants – 0.05. The compound feed itself is produced in the form of granules with a diameter of 4.0-9.5 mm.

Feeding, weight control, and fish maintenance were in full compliance with the fishery management guidelines of Tengryfish LLP.

### Selection and biological research methods for fish

Sampling and organoleptic studies were performed in accordance with the following normative documents: Republic of Kazakhstan Standard 1802-2008, State Standard 7631-85, Republic of Kazakhstan Standard 1803-2008, State Standard 10444.15-94 [21-24]. The fish were gutted, packed in ice, and delivered to the laboratory on the day of slaughter. All analyses were performed the next day. During the organoleptic evaluation, we examined the appearance (surface cleanliness, natural color, flatness of the scales, and presence of external damage), correctness of cutting, consistency (dense, weakened, but not flabby was also accepted), and smell (characteristic of fresh fish).

**Figure-3:**
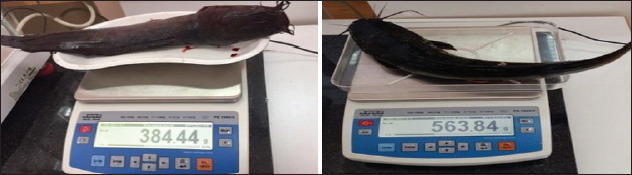
Determination of fish weight.

The absolute length was measured from the end of the snout to the middle of a straight line connecting the ends of the extreme rays of the caudal fin. Body lengths were measured from the top of the snout to the end of the scale covering. The length of the head was measured from the top of the snout to the rear end of the gill cover excluding the membrane. The length of the carcass was measured from the rear edge of the gill cover to the end of the scale covering. The greatest body height was measured as the distance between the highest point of the dorsum (in front of the dorsal fin) and the lowest point of the abdomen. The weight of fish was determined individually on a scale according to the State Standard 1368-2003. Fish Length and Weight [25] (Figure-3).

Appearance, body shape, fins, and fish mouth were examined visually. Color was evaluated on a fresh transverse section at the fleshiest part. Consistency and density were determined over the entire surface of the fish visually or by lightly squeezing the fish with the fingers or by pressing on a cross-section. The state of the fish’s eyes was evaluated using two indicators: The position of the eyes relative to the orbits [above the level of the orbits (convex), at the level of the orbits (flat), lower than the level of the orbits (concave)] and the transparency of the cornea. The condition of the gill covers was characterized by one main (mechanical damage) and two additional (position relative to the gills and color) indicators. The condition of the anal ring and internal organs was determined visually using three indicators: Sharpness of borders, color, and presence of helminths. Smell was determined from the surface of the knife after it had been inserted into the fish’s body between the dorsal fin and the back of the head, near the anal opening from the abdomen toward the spine, to the internal organs through the anal opening, at the sites of wounds and mechanical injuries or into the fleshiest part, as well as by smelling the surface of the gills. The knife was preheated for 1-2 min by immersion in hot water and was washed after being used on each sample.

### Microbiological research methods for fish

The microbiological studies were conducted in the microbiological safety laboratory of the Kazakhstan-Japan Innovation Center at the Kazakh National Agrarian University.

Control tests for the presence of pathogenic microorganisms in fish were carried out in accordance with the requirements of the following regulatory documents: State Standard 10444.15-94, State Standard 31659-2012, and State Standard 31747-2012 [24,26,27].

### Statistical analysis

The quantitative indicators of the research results were processed by statistical analysis using the Microsoft Excel (Microsoft Corporation, U.S.), Statgraphics Plus (Statgraphics Technologies, U.S.) software package.

## Results

### Biological indicators of fish

The first stage of scientific work after setting up the experiment on fish was to study the effect of the feed additive on fish-breeding biological parameters and the physiological state of fish.

The results of studies on the Clarius catfish of the experimental and control groups are presented in Table-3.

**Table-3:** Biological parameters of sharptooth catfish from experimental and control groups bred at the Tengryfish LLP fish breeding enterprise using the feed additive “Ceobalyk” (average values).

No	Parameter	Sharptooth catfish (groups)	

		Control	Experimental
1	Total (absolute) length, cm	41	42
	Body length, cm	35.5	36.5
	Head length, cm	9.3	10
	Carcass length, cm	25	25
	Greatest body height, cm	5.5	5.5
	Greatest body thickness, cm	5.9	6
2	Body weight, g	384.44	563.84
	Appearance	No external mechanical injuries, cuts, disruptions, and cracks; mucous	No external mechanical injuries, cuts, disruptions, and cracks; mucous
3	Body shape	Serpentiform, unchanged
4	Color	Characteristic dark gray color	Characteristic dark gray color
5	Consistency	Elastic, thick, recovers easily after pressing with a finger	Elastic, thick, recovers easily after pressing with a finger
6	Smell	Weak characteristic smell Seethed product was separated from the broth and placed on a plate, the product and the broth were smelled while hot	Weak characteristic smell Seethed product was separated from the broth and placed on a plate, the product and the broth were smelled while hot
7	Mouth	Closed	Closed
8	Eyes	Convex, with clear corneas, with no external damage, characteristic of fresh fish, no abnormality	Convex, with clear corneas, with no external damage, characteristic of fresh fish, no abnormality
9	Fins	Clean, with no external mechanical injuries and laceration	Clean, with no external mechanical injuries and laceration
10	Scales	Absent	Absent
11	Gill cover	Rest firmly against gills, no damage	Rest firmly against gills, no damage
12	Gills	Red, clean, no foreign particles	Red, clean, no foreign particles
13	Anal ring	Pale pink	Pale pink
14	Internal organs and abdominal cavity	Not inflated, intact no internal damage, no parasites found	Not inflated, intact no internal damage, no parasites found
15	Gall bladder	Watery	Watery
16	Color and appearance of caviar	Green	Green

Organoleptic study of the samples of fish that received the feed additive “Ceobalyk” showed that their skin was even, clean, smooth, without bruising, and mechanical damage, and was covered with thin mucus. The scales were solid, shiny, with a pearlescent shade, and were firmly secured.

### Physicochemical results of fish research

**Figure-4:**
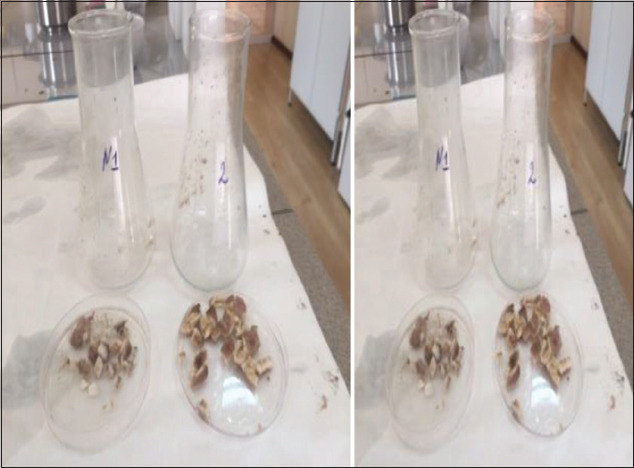
Study of fish smell.

The internal organs were not enlarged, there was no noticeable bruising, and the smell was typical. The musculature was dense, elastic, and resilient; after pressing on the skin with a finger, the depression did not persist. The fish had a characteristic fresh smell (Figure-4).

**Figure-5:**
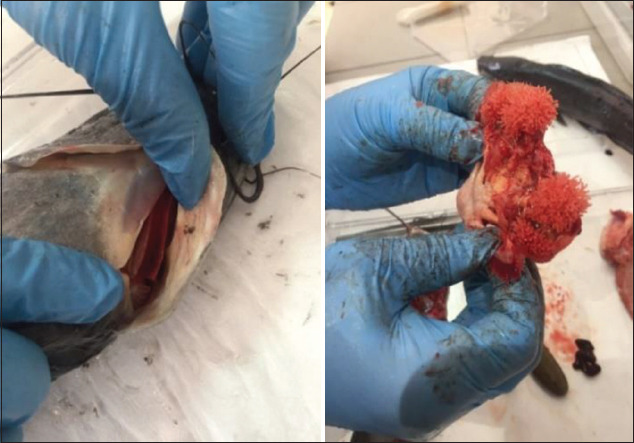
Study of the gill cover and gills.

The eyes were shiny, convex with transparent corneas, with no external damage, characteristic of fresh fish, without pathology. The gills were pale pink, without the smell of decomposition or rotting (Figure-5).

**Figure-6:**
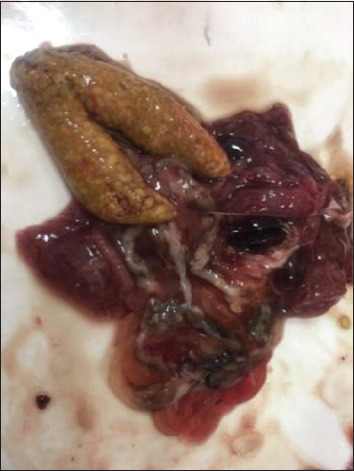
Study of the gallbladder.

**Figure-7:**
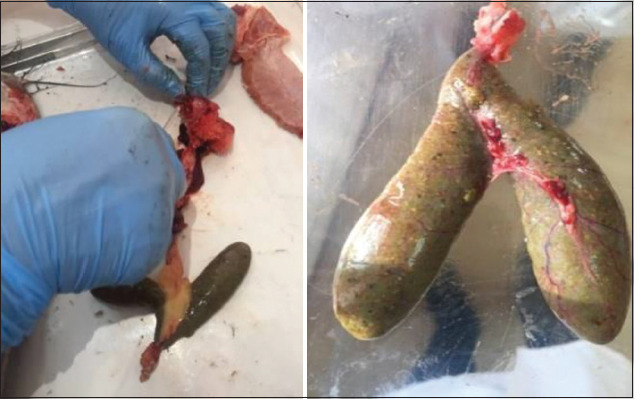
Study of the caviar color and appearance.

When boiled, the broth was transparent and full-flavored. The gallbladders of the fish were watery. The color of the caviar was green and there were no pathological changes (Figures-6 and 7). The results of the studies on the physicochemical parameters of fish samples are shown in Table-3. Table-4 shows that the pH of the catfish meat from the experimental group was in the range from 6.6 to 6.7.

**Table-4:** Changes in the physical-chemical parameters of sharptooth catfish meat with the use of the new feed additive “Ceobalyk.”

Groups	Control	Experimental
Physical-chemical parameters of fish		
Reaction with a 5% solution of copper sulfate	Clewar solution	Clear solution, no flakes
Bacterioscopy	5-7 cocci and bacilli on the surface	4-5 cocci and bacilli on the surface
Amino ammonia nitrogen (mg)	1.23±0.1	1.25±0.2
Peroxidase presence determination	+	+
Hydrogen sulfide presence determination	No reaction, no change	No reaction, no change
Reaction with Nessler’s reagent	Clear solution, no visible turbidity	Clear solution, no visible turbidity
pH	6.7±0.3	6.6±0.1

Studies demonstrated that the fish meat filtrates in the reaction with 5% solution of copper sulfate remained transparent, clean, with no flakes, and no jelly-like precipitate formation in all groups. It was found that the amount of amino ammonia nitrogen in the experimental and control groups averaged 1.24±0.25 mg, which is normal.

During the reaction to determine the presence of peroxidase in the fish samples, the color of the solution turned blue-green and, after 1 min, it turned reddish-brown, which indicates the freshness of the meat samples. The result of the peroxidase test was positive in all experimental groups. The reaction to determine the presence of hydrogen sulfide showed that all samples met sanitary standards, as there was no reaction (the drops placed on meat did not turn dark brown). Determination of the Nessler number showed that the filtrate obtained from fish of all groups was transparent, with no turbidity and yellowing. This confirms that the Nessler number did not exceed 1.0 and thus that all fish samples were fresh.

###  Microbiological results of fish research

Bacterioscopy of impression smears showed that microorganisms were absent from the deep layers in all groups of catfish. Five to seven cocci and rods were found on the surface in the control group and four to five cocci and rods were found in the experimental group.

**Figure-8:**
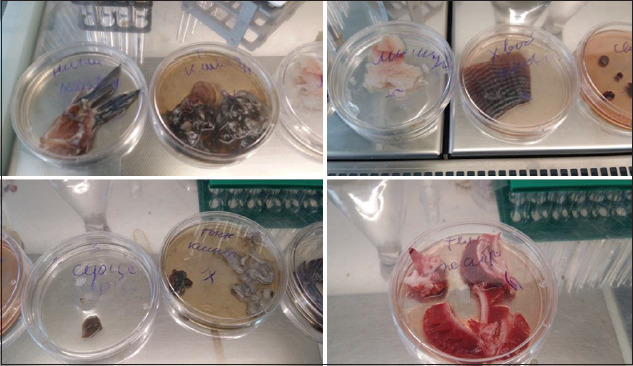
The study of sharptooth catfish microbiological safety.

The results of a bacterial content study (Table-5) and determination of the content of coliforms and pathogenic microorganisms, including salmonella (Table-6), in the samples of control and experimental groups of fish are given below (Figure-8). According to the results, the total viable count values in the fish samples from all groups ranged on average from 1.0×10^4^ to 5.6×10^4^ CFU/g (cm^3^) (Table-5). On average, total viable count values were 2.4×10^4^ CFU/g (cm^3^) in the control group meat samples, 1.1×10^4^ CFU/g (cm^3^) in the experimental group meat samples, 3×10^4^ CFU/g (cm^3^) in the gills of fish from the control group, and 3.2×10^4^ CFU/g (cm^3^) in the gills of fish from the experimental group.

**Table-5:** The results of the bacterial content study (total viable count) of samples of sharptooth catfish (average values).

No.	Organs	Dilution		Results CFU/g (cm^3^)

		10^4^	10^5^	
Control group (n=10)				
1	Gills	48	17	3×10^4^
2	Heart	0	0	1×10^4^
3	Kidneys	0	0	1×10^4^
4	Liver	26	7	1.5×10^4^
5	Stomach	80	44	5.6×10^4^
6	Intestines	201	70	1.2×10^5^
7	Meat	29	23	2.4×10^4^
Experimental group (n=10)				
1	Gills	51	20	3.2×10^4^
2	Heart	30	10	1.8×10^4^
3	Kidneys	40	15	2.5×10^4^
4	Liver	26	7	1.5×10^4^
5	Stomach	80	44	5.6×10^4^
6	Intestines	60	45	4.8×10^4^
7	Meat	18	6	1.1×10^4^

**Table-6:** The results of the determination of the content of coliforms (Kessler medium) and pathogenic microorganisms, including salmonella (magnesium medium).

No.	Organs	Kessler medium color change	Magnesium medium color change
Control group			
1	Gills	Gas+clarification	Turbid straw-yellow
2	Heart	Gas+clarification	Turbid straw-yellow
3	Kidneys	Gas+clarification	Turbid straw-yellow
4	Liver	Gas+clarification	Turbid straw-yellow
5	Stomach	Gas+clarification	Yellow-green
6	Intestines	Gas+clarification	Turbid straw-yellow
7	Meat	Gas+clarification	Turbid straw-yellow
8	Control	No change (dark blue)	No change (dark green)
Experimental group			
1	Gills	Gas+clarification	Yellow-green
2	Heart	Gas+clarification	Yellow-green
3	Kidneys	Gas+clarification	Yellow-green
4	Liver	Gas+clarification	Yellow-green
5	Stomach	Gas+clarification	Yellow-green
6	Intestines	Gas+clarification	Yellow-green
7	Meat	Gas+clarification	Yellow-green
8	Control	No change (dark blue)	No change (dark green)

To study the contents of pathogenic microorganisms, including salmonella, coliforms, listeria, and staphylococci, we used the following media: Peptone buffered, magnesium, Endo, bismuth-sulfite, Giss’s, triple sugar, Fraser’s broth, Palcam agar, saline broth, and staphylococcal agar. Studies of the fish samples revealed no pathogenic microorganisms (including salmonella, listeria, coliforms, and staphylococci).

## Discussion

This experiment demonstrated that the “Ceobalyk” additive introduced into the diet of catfish also affects their external parameters. For example, the body length in the experimental group was significantly greater (by 2.12% on average) than that in the control group. The head length in experimental fish was also greater than that in the control group. In addition, the head width was 1% shorter than that in the control. In turn, we observed increases in the absolute body length and body width, which might indicate that the additive contributed to elongation of the experimental catfish body. The intestinal length was the same in both groups. Analysis of internal indicators demonstrated that sharptooth catfish had good commercial qualities, and the use of the feed additive in the feed did not significantly affect the biology of the fish. Similar results on the effect of this type of feed additive on the growth, development, and efficiency of the use of compound feeds were obtained in a previous study [28], confirming the positive effect of the use of feed additives based on natural zeolite.

Organoleptic studies revealed that the musculature of the fish of the experimental group was dense and elastic; when pressed on the skin with a finger, a depression did not remain. In addition, the smell was characteristic of freshness. Similar results were also obtained by Sharma and Kumar [29]. They showed that higher fish productivity was achieved in ponds treated with zeolite than in the control.

Physicochemical studies also showed that the pH values of fish meat in the experimental and control groups were normal, varying from 6.6 to 6.7. When setting up the reaction with copper sulfate, the reaction was positive; the broth remained clear after the reaction; the meat can be considered of high quality in terms of its freshness. When the sample was cooked, the broth was transparent, with a specific fish-like smell. The peroxidase reaction was positive; during the Nessler reaction, the filtrate was clear, with no yellowing or turbidity. In a study by Skleničková [30], it was proved that the zeolite filter in the fish farming recirculation systems did not have a negative effect on the physiological state of fish. This proves that zeolite is completely harmless and does not have negative effects on the physicochemical properties of living organisms.

During bacterioscopy, a decrease in the number of microbes was observed in a manner dependent on the concentration of CD. In the experimental group of fish, two to three bacteria were observed on the surface, but no bacteria were observed in the deep layers.

In addition, Kuley *et al*. [31] used various doses of natural zeolite to determine the qualitative changes in sardine fillets in vacuum packaging. Zeolite contributed to improve the sensory qualities of sardines, especially to the removal of unpleasant smells. The use of zeolite led to a significant decrease in the total volatility values of basic nitrogen. From this, we can conclude that zeolite improves not only the appearance and smell but also the antimicrobial properties of the product.

Moreover, Wu *et al*. [32] conducted a study to determine the effect of a zeolite feed additive on the intestinal microflora of broilers. The obtained results showed that the zeolite feed additive reduces the number of pathogenic bacteria in the gut of birds.

Natural zeolite can also be used to reduce the levels of biogenic amines and ammonia in products, which are produced by pathogenic bacterial strains. This was proved in work by Özogul *et al*. [33]. The feed additive “Ceobalyk” improves digestion and thus confers better utilization of feed, which, in turn, stimulates the growth and productivity of fish. Moreover, the “Ceobalyk” feed additive contributes to increasing the acidity in the intestine, which prevents the development of pathogenic bacteria and other undesirable intestinal microorganisms [34].

The analysis of all experimental data showed a strong effect of the “Ceobalyk” feed additive on the main fish breeding indicators. The final mass of experimental catfish that received low protein feed was 2.8% higher than that of the control. Such results were obtained in work by Sheikhzadeh *et al*. [35] who showed the protective effect of zeolite composites on the growth, activity of digestive enzymes, and biochemical parameters in rainbow trout. The addition of zeolite to the diet significantly improved growth rates compared with the control diet. Additional zeolite could enhance amylase activity in the intestines of fish. It was shown that the zeolite composite at a concentration of 5 g/kg has the potential to increase the growth indices, the activity of digestive enzymes, and several biochemical indices in rainbow trout.

In addition, water quality should be monitored to ensure fish growth and survival. Zain *et al*. [36] showed that the addition of zeolite is essential for improving water quality and growth indicators of red hybrid tilapia (*Oreochromis* spp.).

## Conclusion

During the development of new and implementation of traditional technologies for preparing various fish products, sanitary conditions of fish raw materials, auxiliary materials, semi-finished products, and fish products should be monitored using microbiological indicators. In all fish samples, indicators such as total viable count and coliform content conformed to hygienic standards. Other types of bacteria were not found in the samples. A study of the biological parameters of fish that received the new feed additive “Ceobalyk” in their feed showed that it is completely safe and does not have negative effects on the physicochemical properties and freshness of fish meat, the physiological state of the fish that received compound feed with the “Ceobalyk” additive was normal, both in the experimental groups and in the control. Based on this, it can be concluded that this feed additive does not cause deviations in the physiological status of fish and can be used in feed.

## Authors’ Contributions

ASA: Conception and design, analysis, and ­interpretation of data. NBS: Revised the manuscript critically. TBA: Drafted the manuscript and revised it critically. ABA: Acquisition of data. ArBA: Acquisition of data. All authors have read and approved the fnal manuscript.
